# Current role of interventional radiology in thyroid nodules

**DOI:** 10.3389/fendo.2024.1405705

**Published:** 2024-09-17

**Authors:** Onur Taydas, Erbil Arik, Omer Faruk Sevinc, Ahmet Burak Kara, Mustafa Ozdemir, Hasret Cengiz, Zulfu Bayhan, Mehmet Halil Ozturk

**Affiliations:** ^1^ Department of Radiology, Faculty of Medicine, Sakarya University, Sakarya, Türkiye; ^2^ Department of Radiology, Faculty of Medicine, Marmara University, İstanbul, Türkiye; ^3^ Department of Radiology, Kocaeli City Hospital, Kocaeli, Türkiye; ^4^ Department of Radiology, Gaziantep City Hospital, Gaziantep, Türkiye; ^5^ Department of Endocrinology and Metabolism, Faculty of Medicine, Sakarya University, Sakarya, Türkiye; ^6^ Department of General Surgery, Faculty of Medicine, Sakarya University, Sakarya, Türkiye

**Keywords:** thyroid nodule, interventional radiology, biopsy, ablation, embolization

## Abstract

Thyroid nodules are a prevalent health issue in society. Interventional radiological methods are successfully applied for both the diagnosis and treatment of nodules. Diagnostically, a fine-needle aspiration biopsy and a core needle biopsy can be performed to ascertain the benign or malignant nature of a lesion. In recent years, imaging-guided percutaneous treatment methods have become popular in the treatment of thyroid nodules. Aspiration, ablation, and embolization are techniques employed in the treatment process. In this study, we aimed to discuss the current role of interventional radiology in the diagnosis and treatment of thyroid nodules, which occupy an important place in clinical practice.

## 1 Introduction

**Figure 1:**
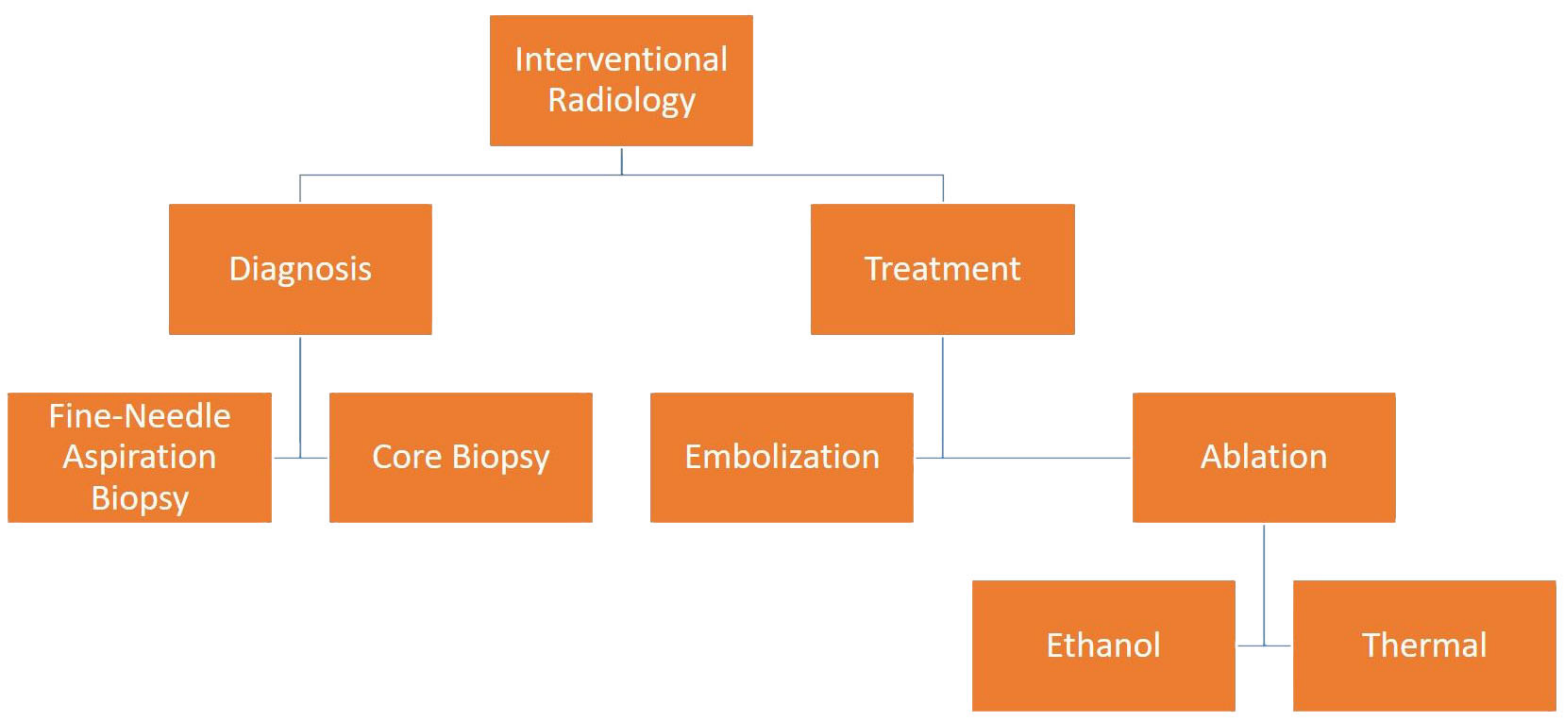
Diagnostic and therapeutic interventional radiological methods for thyroid nodules.

Thyroid nodules are frequently encountered during routine ultrasonography (USG) imaging in the healthy adult population. Although the majority of thyroid nodules are benign, they nonetheless carry a variable risk of malignancy (7-15%) (1). When USG reveals suspicious indications of malignancy, a tissue sample is required to confirm the diagnosis. Fine-needle aspiration biopsy (FNAB) is the primary technique employed to achieve percutaneous tissue diagnosis. Core needle biopsy (CNB) is another method that can be used in the diagnosis of nodules with suspicious USG findings and negative FNAB results (2). Benign thyroid nodules usually do not require treatment; however, treatment is indicated for nodules that have a large size or increase in size during follow-up, causing clinical symptoms or signs, such as compression and hyperthyroidism, or cosmetic problems (3). While the standard treatment for thyroid nodules was surgery in the past, minimally invasive techniques have been developed in recent years. Percutaneous treatments (simple aspiration, ethanol injection, and ablation) and thyroid artery embolization (TAE) are the most common among these techniques (4) (**Figure 1**).

In this review, all the minimally invasive imaging-guided interventional radiologic methods known to date in the diagnosis and treatment of thyroid nodules will be discussed together with their current areas of use. While previous systematic reviews and meta-analyses have addressed specific aspects of this topic, to our knowledge, this review is unique in its comprehensive coverage of all known methods in the existing literature on the subject. By synthesizing the current knowledge on these methods, this review aims to facilitate informed decision-making practitioners by comparing diagnostic and therapeutic methods based on current knowledge and highlighting the most appropriate methods for various clinical scenarios.

## 2 Imaging-guided methods for tissue sampling

### 2.1 Fine-needle aspiration biopsy

FNAB is currently the gold standard in the diagnosis of thyroid nodules. USG follow-up is considered sufficient for nodules smaller than 5 mm in size. For nodules with a diameter of 5-10 mm presenting with suspicious findings on USG, close observation is recommended, taking into account FNAB findings, clinical condition, or the patient’s preference. In these nodules, FNAB is indicated in the presence of a suspicious lymph node, extrathyroidal enlargement, an individual or family history of thyroid cancer, or if the lesion is subcapsular or paratracheal (3). The USG findings of thyroid nodules are divided into three categories: low risk, moderate risk, and high risk. Considering these categories, FNAB indications for lesions of 10 mm and above have been defined as follows (3):

1. ≥10 mm and high-risk findings on USG
2. ≥20 mm and moderate-risk findings on USG
3. ≥20 mm and low-risk findings on USG if
  - there is an increased lesion size during follow-up
  - there is a history of risk
  - surgery or percutaneous ablation is planned

If a hot nodule is detected on scintigraphy, FNAB is not recommended due to the very low probability of the nodule being malignant (5). The presence of a bleeding diathesis or infection at the entry site is considered a contraindication for FNAB (6). The guidelines of the Society of Interventional Radiology (SIR) evaluate FNAB in the low risk category in terms of bleeding and do not routinely recommend the discontinuation of anticoagulant medications before the procedure (6). Performing the procedure under USG guidance enhances the safety of the procedure by providing real-time control of the needle tip. By employing this method, the surrounding vascular structures are protected while obtaining a biopsy sample from the target area (4). The Bethesda classification, updated in 2017, divides cytology results into six categories (7):

1. Non-diagnostic/unsatisfactory
2. Benign
3. Atypia or follicular lesion of undetermined significance
4. Follicular neoplasm (FN)/suspicious for FN
5. Suspicious for malignancy
6. Malignancy

FNAB is performed with the patient placed in the prone position and the neck in hyperextension. Following the cleansing of the neck with alcohol or betadine, local anesthesia is administered. During the procedure, needles with a diameter of 21-27 gauge connected to a 10 cc syringe are typically used. After reaching the target nodule under USG guidance, the needle tip is moved back and forth while applying negative aspiration, and the cell is withdrawn. After the retrieved material is fixed, it is transferred to the pathology laboratory (4).

The complication rate of FNAB is low, with the most well-known complications being cervical hematoma, local infections, and vasovagal syncope. The use of antiseptic solutions, the procedure performed by an experienced operator under USG guidance, and the application of manual compression after the procedure significantly reduce the development of complications (4).

It has been reported that measuring washout thyroglobulin (Tg) levels with FNAB in suspicious lymph nodes in papillary thyroid cancer (PTC) increases the diagnostic power of FNAB in the diagnosis of metastatic lymph nodes. This method also plays a role in identifying recurrences in differentiated thyroid cancers (8). In this technique, following FNAB of the lymph node and the fixation of the obtained material, the biopsy needle is washed one or more times by adding at least 1 ml of saline to the aspiration syringe. The collected material is then sent to the laboratory for Tg measurement (9). However, challenges such as the lack of standardization for the applied technique and the absence of a standard cut-off value for malignant lymph node diagnosis pose obstacles in washout-Tg measurements (8).

On the other hand, fine needle cytology can be performed without syringe connection to the needle, thereby eliminating the need for suction. This technique, also called fine needle non-aspiration biopsy (FNNAB), has been suggested to have advantages over conventional FNAB, such as being easier to perform and causing less hemorrhage (10). There are studies in the literature comparing FNAB and FNNAB. In a meta-analysis published in 2015 by Song et al. examining the results of five separate studies, both techniques were found to be equal in evaluating thyroid nodules (11). Sasikumar et al. reported that FNNAB yielded better results due to the better quality of cellularity in the specimens and reduced blood products (12). In a prospective study, Heidar et al. concluded that the sample quality obtained through FNNAB was superior. In addition, the authors emphasized the benefits of FNNAB for both the operator and the patient, as it is easier to apply and more comfortable for the patient due to the absence of a syringe (10).

### 2.2 Core needle biopsy

FNAB is widely recognized as the safest and first-line diagnostic method in the diagnosis of thyroid nodules. However, FNAB yields non-diagnostic or indeterminate cytology results (Bethesda categories 3-5) in approximately 30% of cases. In these clinical cases, CNB serves as an alternative diagnostic method to repeat FNAB or surgery (13). Among the most important reasons for this is that repeat FNAB in nodules with initially non-diagnostic or indeterminate FNAB results produces inconclusive results again in almost half of the cases. Another reason is that the pathology result is significantly benign in nodules that have indeterminate cytology results and are referred to surgery (14). It has also been suggested that CNB may be the first-line method for diagnosis in nodules with suspicious sonographic features (15).

**Figure 2:**
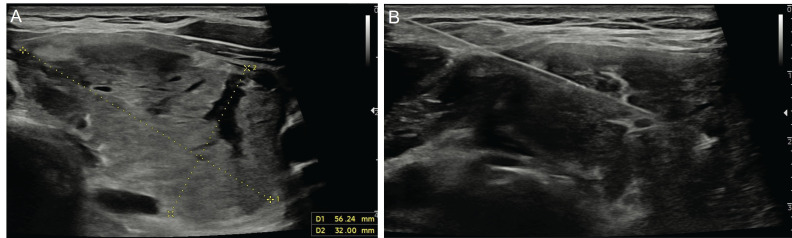
Ultrasonography image of a 35-year-old male patient shows a 56x32-mm nodule **(A)** located in the left lobe of the thyroid gland after three negative fine-needle aspiration biopsy results. The patient underwent a core needle biopsy with a 20G-10cm co-axial semi-automatic biopsy system (Geotek, Turkiye) **(B)** and was diagnosed with follicular neoplasia.

In the literature, studies have shown that the rate of inconclusive results (non-diagnostic and Bethesda category 3) of CNB is lower in thyroid nodules with inconclusive FNAB results when compared to repeat FNAB (16). CNB has certain advantages over FNAB, such as the ability to obtain a larger tissue sample and preserve the cellular architecture (**Figure 2**). This facilitates histopathological evaluation and, if necessary, allows for immunohistochemical tests to be performed (13). In addition, the diagnostic accuracy of FNAB decreases in follicular lesions (16). A previous study showed that CNB was diagnostic in 90% of cases with inconclusive FNAB results (17). The same study reported that CNB had 100% specificity and positive predictive value in the diagnosis of malignant nodules, 97.5% sensitivity in predicting nodules that required surgery, and 98.6% negative predictive value in identifying benign nodules. It has been suggested that the lower rate of indeterminate cytology results in CNB than in FNAB may be due to the fact that histological samples considered to be the follicular variants of PTC in the former are often evaluated as atypia of undetermined significance or follicular lesions (Bethesda category 3) in the latter (18).

A study showed that among nodules for which the initial CNB result is evaluated as insufficient, the malignancy rate was very low (1.3%) in cases of repeat CNB. Therefore, the authors initiated a discussion over whether nodules with insufficient CNB results could be treated as benign nodules (14).

There is not yet consensus on a classification method for CNB, similar to the Bethesda system used in FNAB. In 2015, the Korean Endocrine Pathology Thyroid Core Needle Biopsy Study Group published a classification based on the Bethesda system, consisting of six categories (19). In 2017, the same study group published “Core Needle Biopsy of the Thyroid: 2016 Consensus Statement and Recommendations from Korean Society of Thyroid Radiology”, in which they merged Bethesda categories 3 and 4 into the indeterminate category, further divided into three subtypes (20). Paja et al. proposed that a categorization similar to the simplified Bethesda system, as previously recommended by Walts et al. for FNAB, could also be employed for CNB. Accordingly, the authors discussed the CNB results in the following four categories: non-diagnostic, benign, follicular lesion, and malignant (14, 21).

USG-guided CNB is performed with the patient placed in the supine position and the neck in hyperextension, as in FNAB. After determining the appropriate access to reach the nodule, local anesthesia is administered to the route through which the needle will pass. It has been stated that the most appropriate technique for entry is the trans-isthmic approach (16). During the procedure, fully automatic or semi-automatic needles with a diameter of 18-21 gauge are utilized. Although fully automatic needles are more effective for calcified nodules, they are more likely to damage tissue or vascular structures. Semi-automatic needles are regarded as safer due to the inner needle being pushed manually (16). It is important to monitor the route and tip of the needle throughout the procedure, particularly to prevent passing through areas with large veins. There is a lack of consensus regarding the number of tissue samples to be taken and which part of the nodule should be biopsied. It is generally recommended to take at least two tissue samples. It is important to consider that taking multiple biopsies may increase the risk of complications. If multiple biopsies are to be performed, the use of a coaxial needle is recommended (16). Many studies suggest that the tissue sample taken should encompass the inside of the nodule, the nodule-parenchyma border, and the normal parenchyma (16, 20). Manual compression should be performed after the procedure. In previous publications, the duration of compression varies between 5 minutes and 20-30 minutes (14, 16, 17).

CNB is a safe, well-tolerated method with a low complication rate when performed by experienced operators (16). The complication rate is reported to be comparable to that of FNAB, but post-procedure pain is slightly more severe and lasts longer (14). In the available literature on CNB, complications have been described as hematoma, voice change, infection, hemoptysis, edema, vasovagal reaction, and dysphagia (16, 20). A single-center large series published in 2017 and a meta-analysis published in 2018 reported the rate of major complications to be below 0.1% (22, 23). The former also noted that the total rate of minor complications was 0.79% (23).

## 3 Minimal invasive approaches for treatment

In recent years, imaging-guided percutaneous treatment methods and thyroid artery embolization (TAE) have gained popularity for the treatment of thyroid nodules, mostly due to the risks associated with surgery and radioactive iodine therapy. Percutaneous treatments include simple aspiration, percutaneous ethanol injection (PEI), and thermal ablation (TA). Among the thermal ablation methods are laser ablation (LA), radiofrequency ablation (RFA), and microwave ablation (MWA) (24). Percutaneous methods are generally performed under local anesthesia in an ambulatory setting. Their efficacy has been proven in the treatment of benign, symptomatic, or autonomous functional thyroid nodules (AFTN). Although they are used in the treatment of primary or recurrent PTC, a sufficient evidence level has not yet been reached (25). TAE is an embolization procedure performed after the super-selective catheterization of the thyroid arteries and basically aims to reduce the size of the thyroid nodule (26).

### 3.1 Simple aspiration

In cystic and predominantly cystic nodules, the fluid content is drained through a procedure known as simple aspiration. Predominantly cystic nodules are mostly benign nodules containing more than 50% fluid component. These nodules may need to be treated because they can cause compression or cosmetic problems. Simple aspiration is the first step in the treatment of cystic and predominantly cystic nodules (25). When aspiration is performed without the injection of sclerosing material, a high rate of fluid accumulation (95-98%) is observed in the cyst (27). Nevertheless, aspiration is still a feasible method for the treatment of cystic nodules due to its advantages, including low procedure-related risks, the ability to confirm the benign nature of the nodule, and the provision of cytological information prior to more definitive treatment options, such as ethanol injection, ablation, and surgery (25).

### 3.2 Percutaneous ethanol injection

**Figure 3:**
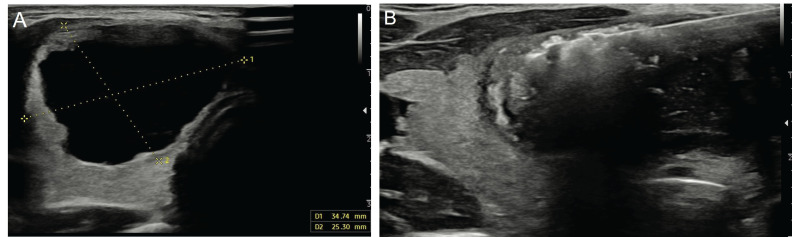
Ultrasonography image of a 28-year-old female patient shows a pure cystic nodule **(A)** measuring 34x25 mm in the right lobe of the thyroid gland. The patient underwent ethanol ablation **(B)**.

PEI is the first-line method in the treatment of predominantly cystic nodules that recur after aspiration and have benign cytology results (**Figure 3**). Given that the probability of malignancy is less than 1% in pure cystic nodules, PEI followed by simple aspiration may be the first-line treatment without obtaining cytology samples. Technically, after aspiration of almost the entire cyst content, ethanol (95-99%) is injected into the cavity at a rate of 30-50% of the aspirated amount (4). This aims to prevent the development of reactive fibrosis in the cyst epithelium after ethanol injection and, thus, the re-accumulation of fluid in the cyst (25). It has been shown that the PEI procedure is effective in 85-90% of pure cystic nodules and 60-90% of predominantly cystic nodules (28). A previous study stated that the most important factor affecting the success of the PEI procedure was the aspiration of the entire cyst (27). Another study reported that a solid component ratio above 20% in predominantly cystic nodules reduced the success of the procedure (29).

The use of PEI, which is commonly employed in the treatment of cystic and mostly cystic nodules, has been compared in the literature with RFA, which is used for similar indications in the treatment of TN. Studies in this field have demonstrated that the success rate of treatment and the rate of complications are similar. Furthermore, research indicates that PEI has certain advantages, such as the ease of application, low cost, and reduced pain perception (30–32) (**Table 1**).

**Table 1:** Comparison of the use of PEI and RFA in the treatment of cystic and predominant cystic nodules.

Study	Cystic component of the nodule	VRR*****(Volume reduction rate)	Advantages of PEI	Advantages of RFA	Complications
**Sung et al.** (30)	>90%	No difference	Less expensive Simpler to perform Lower number of treatment session	NI****	No serious complication in both group
**Baek et al.** (31)	>50% and <90%	No difference	Less expensive Simpler Lesser tendency to experience pain	NI	No significant difference, but only 1 major complication*** in EA group
**Nguyen et al.** (32)	Pure cystic and predominantly cystic**	Similar in predominantly cystic, but higher in RFA for pure cystic group	NI	Better therapeutic success rate* (90.9% vs 52.9%)	No major or minor complication in both group

*therapeutic success defined as if VRR >50%.

**unknown ratio of cystic component.

***transient voice change.

****NI, not informed.

*****VRR=[initial volume - residual volume]/initial volume).

PEI has been utilized in the treatment of AFTNs since the 1990s. The aim of treatment is to shrink the nodule and normalize thyroid function and scintigraphy findings (functional ablation) (33, 34). It has been shown that treatment success is higher in non-toxic and pre-toxic AFTNs and in nodules with a volume of less than 15 ml (35). Recently, Sharma et al. introduced the vascularity-targeted ethanol injection technique, which involves the power Doppler mapping of the blood vessels within the nodule and then injecting ethanol into these vascular structures. This technique has been shown to reduce the amount of ethanol (0.45 ml/ml nodule volume) and the number of injections required for successful treatment compared to the conventional method (36). Although the risk of malignancy is low in AFTNs, it is recommended to confirm that the nodule has benign cytology at least once before performing PEI (25).

Recurrent papillary thyroid cancer (RPTC) constitutes another indication for PEI in selected cases. The decrease in the lowest detectable serum thyroglobulin level and the use of high-resolution ultrasonography have facilitated the detection of cervical recurrence in patients during follow-up. The aim of treatment is to shrink or eliminate the affected lymph node, reduce vascularity, and decrease the serum thyroglobulin level. USG-guided ablation can be performed even on metastatic lymph nodes with a diameter of 2-3 mm (37, 38). Although surgery is recommended as the first-line treatment for RPTC, there is a scarcity of studies comparing surgical and PEI methods. In a retrospective study published by Tofé et al. in 2023, it was shown that the success of treatment in PEI was similar to surgery, and the complication rate was low. The authors also noted that the rate of patients requiring radioactive iodine (RAI) after the procedure was lower in PEI than in surgery (39). A systematic review published in 2015, including the results of 27 studies, found that treatment success was higher for surgery, and post-treatment recurrence was similar between the PEI and surgery groups. The authors emphasized that PEI was a good treatment alternative for patients who were either unsuitable for surgery or unwilling to undergo surgery in the treatment of RPTC (40). The literature also indicates a significant cost difference between PEI and neck dissection, which is the surgical treatment applied in RPTC (25). In a meta-analysis comparing the efficacy and safety of PEI and RFA in the treatment of RPTC, it was reported that the complete disappearance rate was higher and the recurrence rate was lower in RFA, albeit without a statistically significant difference. The same study demonstrated that the serum thyroglobulin level decreased more significantly in PEI (41).

While PEI is generally regarded as a safe treatment, it has been associated with certain uncommon complications, including localized pain, dizziness, local hematoma, and recurrent nerve palsy and periglandular fibrosis due to ethanol extravasation around the nodule. More serious and rare complications include ethanol-related larynx or skin necrosis, Graves’ disease, Graves’ orbitopathy, and Horner syndrome (4). Additionally, a case of Plummer adenoma has been documented in the literature (42).

Due to the possible alcohol-related complications in PEI and the possibility of recurrence, especially in cystic nodules, lauromacrogol (polyoxyethylene lauryl ether) injection (LE) has been introduced as a new alternative to alcohol. This sclerosing agent, which has been used in the treatment of esophageal variceal bleeding, varicose veins, and even visceral cysts, is rarely used in cystic thyroid nodules (43). During the procedure, after aspirating the cystic content of the nodule under imaging guidance as in PEI, lauromacrogol is administered into the cavity at 30-50% of the aspirated amount. It has been recommended that the total amount of lauromacrogol given should not exceed 20 ml and the needle should be withdrawn with negative aspiration to prevent leakage from the nodule (43, 44). Dong et al. reported that LE showed more effective treatment performance in purely cystic nodules compared to purely cystic nodules, similar to PEI. In the same study, a comparison was made with the results reported in the literature regarding PEI, and it was emphasized that the treatment success rate in LE was similar to PEI, while the complication rate was lower (43). Another study by Dong et al. showed that high vascularity within the solid component (grade 2-3 intranodular vascularity) in largely cystic nodules was a risk factor for ineffective treatment in LE (44). Gao et al. similarly reported that treatment efficacy decreased in vascular predominant cystic nodules (45). In a meta-analysis published in 2021, Yang et al. investigated the efficacy of PEI and demonstrated that the success rates of PEI and LE were similar, but the cost was higher in LE (46). Min et al. compared the efficacy of LE and MWA in largely cystic nodules and concluded that LE was more advantageous due to its lower cost and shorter hospitalization period, despite its similar efficacy (47).

### 3.3 Thermal ablation

**Figure 4:**
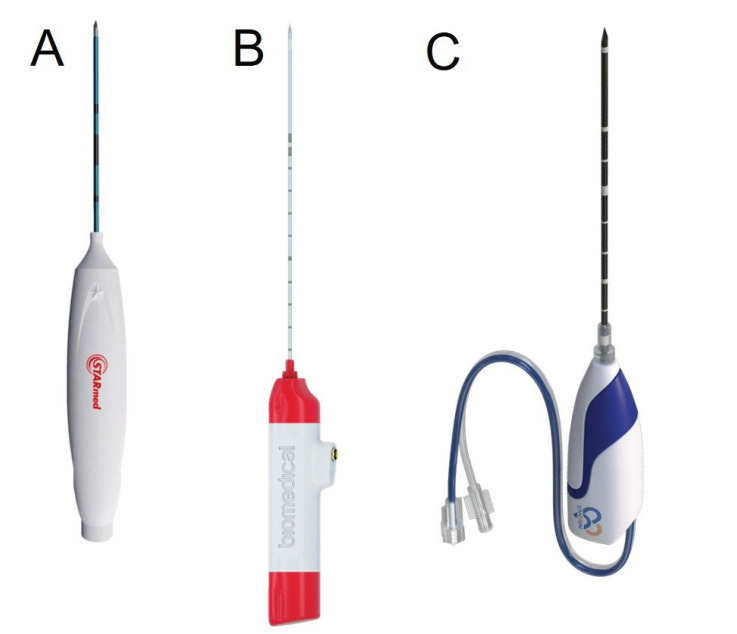
Radiofrequency (Starmed, Republic of Korea) **(A)**, uncooled microwave (TATO, Terumo, Japan) **(B)**, and cooled microwave (Canyon, China) **(C)** ablation probes.

**Figure 5:**
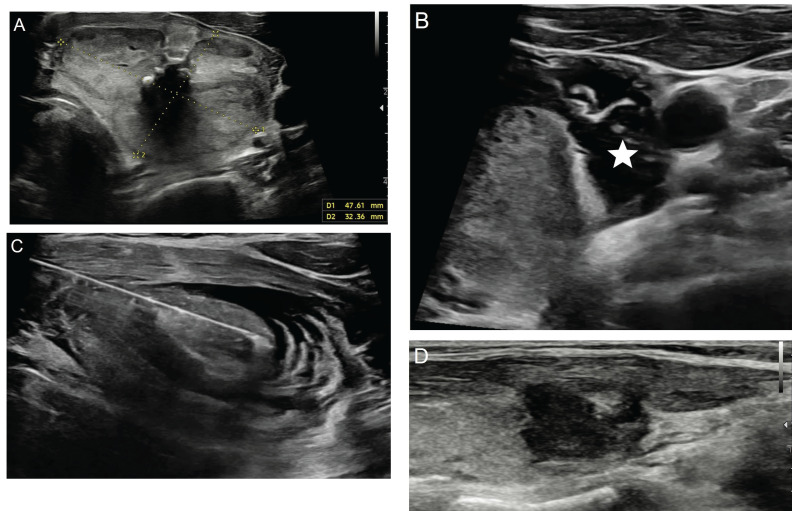
Ultrasonography image of a 57-year-old male patient show a benign solid nodule **(A)** measuring 47x32 mm in the left lobe of the thyroid gland, as confirmed by two separate pathological examinations. The patient underwent hydrodissection (**B**, asterix) followed by percutaneous microwave ablation with the trans-isthmic approach **(C)** using 17G uncooled system (TATO, Terumo, Japan). In the first year control ultrasonography image, a significant loss in size and echogenicity is observed in the nodule **(D)**.

Considering the risks and complications associated with surgery and RAI in the treatment of thyroid nodules, TA has emerged as an alternative to conventional treatment options. TA methods technically utilize non-ionizing electromagnetic energy to induce heat-mediated coagulation necrosis in the target tissue. TA is usually performed under local anesthesia or conscious sedation with USG guidance. The procedure is performed percutaneously by placing optical fibers in LA and probes in RFA and MWA into the nodule (**Figure 4**). The most commonly used basic methods for TA are the trans-isthmic approach and the moving-shoot technique. In the trans-isthmic approach, the isthmus is first entered with an optic fiber or probe, which is then guided laterally from the midline toward the target nodule (**Figure 5**). Entering the nodule with a trans-isthmic approach has certain advantages. First, it minimizes the risk of thermal damage that may occur in the recurrent laryngeal nerve located between the trachea and the thyroid gland. Second, it prevents the hot ablation fluid from escaping into the peritracheal area as a result of the isthmus parenchyma serving as a barrier. Lastly, it ensures the stability of the probe throughout the procedure (48). During the ablation of thyroid nodules, it is recommended to gradually withdraw the active end of the fiber optic or probe, rather than keeping it in a fixed position, as would be the case in the ablation of other organs. In this technique, called “moving shoot”, ablation starts from the farthest area. For safety reasons, it is recommended to leave a distance of 1-2 mm between the distal end of the electrode and the margin of the nodule. Using this technique, the thyroid nodule is divided into ablation units, and ablation is applied to each unit for a few seconds while the electrode is being withdrawn. The primary objective here is to create a sufficient ablation zone and prevent complications that may arise from the ellipsoidal shape of the thyroid nodule and the presence of critical structures around the thyroid gland (24, 48).

In addition to the basic methods described above, several techniques have been introduced, including the Doppler USG-guided ablation of vascular structures, zigzag moving technique, the hydrodissection (HD) method, and the alienate maneuver, to increase treatment success and reduce complications. The artery-first ablation technique involves performing ablation by visualizing the feeding artery of the nodule with Doppler USG, especially in hypervascular nodules. Arterial ablation aims to create ischemic necrosis and reduce the heat-sink effect and hemorrhage. In addition, it has been shown that a technique called “marginal venous ablation”, which involves the ablation of the perinodular veins, can prevent the regrowth of the nodule. If arterial and venous ablations are to be performed together, it is recommended to perform arterial ablation first (49). In the zigzag moving technique, the nodule is divided into ablation units from deep to superficial during the planning of the procedure. After entering the nodule through the trans-isthmic approach, the ablation procedure is initiated from the farthest and deepest part of the nodule and continues with the superficial part of the nodule with gradually less inclined angles, without exiting the nodule. Thus, the ablation of different parts of the nodule is achieved with a single puncture (49). The HD method is used to separate nodules adjacent to risky structures or located in risky areas (recurrent laryngeal nerve, esophagus, vagus nerve, sympathetic ganglion, or areas close to the skin or trachea) from the surrounding tissue and to identify the ablation zone (24). There are different techniques for HD. The perithyroidal lidocaine injection, also known as the anterolateral approach, aims to protect the middle cervical sympathetic ganglion and vagus nerve, especially during the ablation of lateral nodules. Furthermore, this approach minimizes the pain experienced during the procedure. However, care must be taken to ensure that the lidocaine applied does not pass into the peritracheal area to prevent voice changes. The pre-tracheal approach is employed specifically for isthmus lesions. In the danger triangle approach, HD is applied from the posterior of the isthmus (pre-tracheal area) to the space around the danger triangle, with the aim of protecting the esophagus and recurrent laryngeal nerve. In the posterior approach, the solution is applied between the thyroid tissue and the retropharyngeal space by entering through the trans-isthmic or lateral cervical method. This approach is especially recommended for deep-located thyroid nodules located in the right lobe. In general, it is recommended to add 5% dextrose to the HD solution and to use a solution volume greater than 40 ml. Dextrose is used instead of normal saline since the latter is an ionic solution that conducts electricity and has the potential to cause damage to surrounding structures by creating frictional heat (49). In the alienate maneuver, once the needle is delivered to the deepest part of the nodule with the trans-isthmic approach, upward pressure is applied to move the deep nodule in the anterolateral direction. To prevent the needle from dislodging while exerting upward pressure, it is recommended to use larger antennas or probes (16-18 gauge) (49).

TA methods offer several advantages over surgery, which is the conventional treatment for thyroid nodules. First, TA procedures do not require hospitalization or general anesthesia. Second, they have low rates of major complications. Third, they reduce the likelihood of developing hypothyroidism after the procedure. Lastly, they do not result in the formation of cervical scars (50, 51). The efficacy of TA methods is typically assessed using the volume reduction rate (VRR) [initial volume - residual volume]/initial volume). In the literature, despite the results varying depending on the patient series and the technique used, for a single ablation session for all methods, the VRR has been reported as 48-85% and the complication rate as 1-3% (24). In a meta-analysis including approximately five-year patient follow-up of 939 patients who underwent TA in five different studies, the VRR was determined to be approximately 75% at the end of the fifth year. In the same study, the rates of the regrowth of the nodule after the procedure, the need for a second treatment (TA, surgery, or RAI), and complications (minor and major) were reported to be 10.6%, 9.6%, and 7.7%, respectively (50).

LA is based on the principle that laser light is absorbed by the target tissue using optical fibers, resulting in heating and coagulation necrosis. A multisource laser system with a wavelength of 1,064 nm and 21-G introducer needles are generally used for LA. If more than one needle is used for the procedure, the needles are spaced approximately 1 cm apart. Each introducer needle sheath contains a 300-µm-thick bare optical fiber, and the sheath is retracted approximately 5 mm before ablation is started, allowing the optical fiber to contact the thyroid tissue. An important advantage is that it provides a predictable ablation zone with defined borders. However, a disadvantage arises when more than two needles are required for the treatment of larger lesions (52). In a review published by Kuo et al., the VRR in LA ranged between 57% and 95% at the end of 12 months of follow-up (52). In a prospective study undertaken by Gambelunghe et al. through a 10-year follow-up of 171 patients who underwent LA, the VRR was 59% at the end of the follow-up period. In the same study, while no nodular regrowth was observed in any patient at the end of the first year, this rate was 4.7% at the end of the seventh year (53).

RFA uses electric current to induce tissue heating and thus coagulation necrosis in the target nodule. RFA operates through monopolar or bipolar systems. Monopolar systems are more commonly used and require a dispersion or ground electrode to complete the circuit. In bipolar systems, the current oscillates between two electrodes on the same or different needles. In RFA, 18-G probes with a length of 7-10 cm and an active tip of 5-15 mm are generally used. In bipolar systems, 16-G probes with an active tip ranging from 9-40 mm are preferred. In order to prevent heating at the active end of the electrodes, there may be an internal cooling system in the electrode (54). Studies comparing RFA with other ablation methods one-to-one have shown certain advantages, such as higher VRR, lower recurrence, and major complication rates, which will be detailed in the following paragraphs with comparative tables. However, RFA also has some disadvantages related to the principle of physics. When the tissue temperature exceeds 100°C, vaporization and carbonization occur. In addition, heterogeneous nodules exhibit varying thermal and electrical conduction, and the blood circulation, known as “heat-sink”, cools the tissue, which may reduce the effect of ablation (55). In a systematic review of 17 studies published in 2021, the VRR for benign thyroid nodules was 67%-75% at the 12^th^-month follow-up (56). The results of a meta-analysis emphasized that nodule size might be an important determinant for VRR, and it was shown that the VRR was 75% in nodules with a size of <30 ml and 63% in nodules with a size of >30 ml over a 12-month follow-up (57). According to the results of another meta-analysis of 10 studies investigating the effect of RFA in AFTN, the VRR was reported in the range of 50%-85%. In the same study, the normalization of thyroid function was in a wide range of 21%-94%, which may be due to the variability of nodule sizes across the studies (58). However, subsequent studies have not reached a consensus on the relationship between nodule size and the normalization of thyroid function (54).

In MWA, electromagnetic waves in the microwave energy spectrum (300 MHz-300 GHz) are used to produce frictional heating of polar molecules in the target tissue, resulting in tissue necrosis. Probes ranging from 16 to 18 G are used for ablation, and a cooling system may be available as in RFA (52). MWA has potential benefits, including the ability to reach a larger ablation zone in a shorter time and provide more homogeneous ablation due to its physical principle. This makes MWA advantageous in the treatment of larger nodules. In addition, the more homogeneous and predictable ablation zone provides an advantage in the treatment of nodules adjacent to critical structures. However, the rapid temperature increase (150°C and above) that occurs in the central part of the nodule can easily lead to carbonization, which has been associated with a lower VRR compared to RFA (<110°C) in recent studies and meta-analyses (59). The microwave output power during MWA is known to be a factor affecting carbonization. Some studies have concluded that VRR is similar to RFA when the output power is reduced appropriately (59). According to the results of seven studies in a meta-analysis, the VRR was found to be 63% after 12 months of follow-up (60). In another meta-analysis comparing MWA with RFA, the VRR was found to be 80% after 12 months of follow-up (61). In a recent meta-analysis published in 2024, according to the data obtained from five studies, the VRR varied between 65% and 82.5% after 12 months of follow-up (62).

High-intensity focused ultrasound (HIFU) is another thermal ablation method used in the treatment of benign thyroid nodules. It emerged later than other thermal ablation methods for TN. Its physical principle is to generate mechanical and thermal energy through high-energy waves targeting specific tissue (63). The main advantage of HIFU is that it is less invasive as it does not require needle access (64). The use of HIFU in TN was initially observed in 2011, and since then, there have been subsequent studies investigating its therapeutic potential. However, there is no current guideline for the use of HIFU in TN, and there is also a lack of sufficient research demonstrating the success of this technique.

Studies comparing LA and RFA indicate that while these two treatments have similar efficacy, RFA surpasses LA in terms of the risk of complications and the reduction in nodule volume (24) (**Table 2**). A randomized controlled study (LARA II), which compared the 12-month follow-up results of patients who underwent LA and RFA, reported that the VRR was higher in RFA (65). The comparison of RFA with MWA reveals similar levels of treatment efficacy and safety. When compared in terms of complication rates, recurrent nerve paralysis seems to be slightly more common in MWA, although there is no significant difference (24) (**Table 3**). In a meta-analysis published by Guo et al., incorporating the results of five separate studies, the VRR was shown to be higher in RFA than in MWA (61). A similar conclusion was reached in a study conducted by Cerit et al., evaluating 80 patients who underwent TA (59).

**Table 2:** Advantages of LA and RFA over each other.

LA	RFA
Less expensive Feasible in patients with pacemarker	Higher VRR at follow-up Better technical success rate Lower risk of recurrence

**Table 3:** Advantages of MWA and RFA over each other.

MWA	RFA
General advantages based on the physics (larger ablation zone, more homogeneous distribution of heat, and less heat loss)	Higher VRR at follow-up Lower risk of major complication

The indications for TA in benign nodules are comparable to those for the treatment of benign thyroid nodules and can be generally divided into two groups: esthetic and functional. Therefore, nodules that grow or have reached a significant size and/or induce hyperthyroidism should be treated. Toxic multinodular goiter (MNG), MNG without a dominant nodule, and Graves’ disease have been listed as contraindications to TA (24). It is recommended to perform FNAB twice before ablation to confirm that the nodule is benign (24).

Recently, TA has begun to be used for indications beyond those for benign thyroid nodules. This has revolutionized the approach to the percutaneous treatment of PTC, RPTC, and FN. However, since TA methods are very new, their use remains controversial due to the limited number of studies and short patient follow-up periods. In the literature, these methods have been reported to be effective and safe in the treatment of papillary thyroid microcarcinoma. Most studies included low-risk nodules that did not show aggressive clinical or sonographic findings and showed that lesions disappeared completely or almost completely with RFA. There are also studies demonstrating the efficacy of TA methods in the treatment of larger nodules (T1b and T2) and suggesting that they may be an alternative to surgical treatment in patients who are not suitable for surgery or do not want to undergo surgery (25, 66). Following the use of TA procedures to treat PTC, sonographic follow-up is recommended at regular intervals (24). The literature also provides corroborating evidence for the use of TA methods in the management of recurrent thyroid cancers. In a study conducted by Choi et al., the results of surgery and RFA were compared in patients with RPTC, and although recurrence-free survival was similar between the two groups, complications were found to be more common in surgery (67). In another study undertaken by Chung et al., it was stated that 91% of RPTC foci disappeared completely with RFA, and no locoregional recurrence or distant metastasis was observed in 66% of the patients during the 80-month follow-up period (68). A meta-analysis published by Chung et al., covering 24 studies, showed that the rate of complications associated with RFA was higher in the treatment of RPTC compared to that of benign nodules, but the authors emphasized that the use of RFA was still safe and effective (69). Although the use of RFA in FN has been raised for discussion, its implementation remains a matter of debate. Surgery is the standard treatment as it shows capsular and vascular invasion, which helps differentiate between benign FN and follicular thyroid cancer. Nevertheless, since FNs are largely benign, there has been a shift in focus toward the use of minimally invasive methods such as RFA. In a study conducted by Dobrinja et al., which included six nodules larger than 20 cc, all patients underwent surgery during follow-up. The authors suggested that RFA might delay surgical treatment and trigger neoplastic transformation in follicular thyroid cancer (70). In contrast, Ha et al., who evaluated 10 nodules, reported that eight of these nodules disappeared completely, there was an average of 99.5% volume reduction in the nodules, and no recurrence developed during the five-year follow-up (71). Given that the malignancy rates for FN are approximately 31% in nodules above 4 cm and 13% in smaller nodules, it is plausible to attribute this discrepancy to the small size of the nodules included in the study by Ha et al. (25, 72). It is clear that additional scientific data is needed to support the use of TA methods in the treatment of FN.

As previously discussed, TA-related complications occur at an acceptable rate. The types of complications observed with all ablation methods are largely similar. Minor complications are localized pain and hematoma, infection, and skin burn, while major complications are voice changes due to recurrent nerve damage, nodule rupture, and thyroid function changes (4, 73).

In the last section on TA methods, technical and technological developments, current problems and promising issues will be discussed. TA applied to benign thyroid nodules is an area of current interest and a subject of extensive research. FNAB is performed before the procedure to determine whether a thyroid nodule is benign. However, since the sensitivity and specificity of FNAB is not 100% in showing that the nodule is benign, there is also the possibility of ablation of malignant thyroid nodules. Therefore, the long-term follow-up of the patient after TA, especially in solid nodules, is very important for the detection of possible malignant nodule recurrences. Although there are many publications in the literature on all TA methods, we often observe follow-up results spanning a duration of up to 12 months. Therefore, we consider it crucial to publish and increase the number of studies including longer-term patient follow-up data.

In recent years, the use of fusion imaging, virtual navigation, and contrast-enhanced ultrasound have been introduced to increase the efficacy of TA procedures and reduce complications. Contrast-enhanced ultrasound has the potential to be useful in defining the target nodule before the procedure and better targeting the nodule during the procedure. In addition, since it allows for the evaluation of whether there is residual viable tissue immediately after the procedure, it is considered that the use of contrast-enhanced ultrasound in conjunction with investigations in this field will contribute to the prediction of VRR in long-term follow-up. The use of fusion imaging and virtual navigation is another current issue. During US-guided TA, microbubble formation may adversely affect the visibility of the nodule. The use of these technologies allows safer ablation with better navigation, especially in the ablation of nodules adjacent to critical structures (74). The previously mentioned peri-procedural techniques, some of which are very new, aim to increase the efficiency and safety of the procedure in nodules adjacent to critical structures. In addition, the needles used in TA procedures are also the focus of technological developments. These include needle diameter reduction to increase patient comfort, internal cooling systems to prevent probe heating, and innovations to improve the ablation zone. With current and further developments, the efficacy and safety of procedures may increase.

The many uses of TA modalities have been discussed in detail in previous chapters. To summarize, indications for the use of TA as first-line therapy for benign thyroid nodules are established by national societies, international guidelines, and consensus statements. However, there is still a lack of agreement and clear recommendations for the use of TA in AFTN and primary or recurrent thyroid cancers. Therefore, it is critical to increase the number of publications on TA treatments to be applied in these clinical situations in order to increase both the scientific evidence value and the attention and awareness of other clinicians.

### 3.4 Thyroidal artery embolization

TAE refers to the selective catheterization and embolization of thyroid arteries following percutaneous arterial access. Although this procedure is usually performed via the transfemoral route, it has been suggested that the transradial technique can also be employed (75). After arterial puncture and vascular sheath placement, superior and/or inferior thyroid arteries are selectively catheterized using wire and catheter manipulations. Particle embolization is carried out through a microcatheter by including up to three of the four feeding arteries (two pairs), depending on the specific indication of the procedure (embolization of the entire gland or nodule embolization). In the literature, polyvinyl alcohol (PVA) particles with sizes varying between 150 and 700 µm are generally used as embolizing particles. Following particle embolization, coil embolization can also be undertaken to block the proximal feeding arteries (26, 75).

The first clinical study on TAE was published in 1994, and this method has gained significant popularity in the treatment of thyroid diseases since the early 2000s (76). The relevant literature indicates that, until recent years, TAE was generally used in the treatment of Graves’ disease (diffuse toxic goiter). The main success of TAE in Graves’ disease lies in its ability to effectively address the hyperthyroidism manifestation and reduce thyroid gland volume. The reduction in gland volume achieved by TAE prompted consideration of its potential application in the treatment of thyroid nodules in suitable patients. Studies highlight the advantages of TAE over other standard or minimally invasive treatments for thyroid nodules. The subsequent paragraphs provide a comprehensive analysis of these advantages. The uses of TAE in thyroid diseases other than thyroid nodules (solitary or multiple nodules) can be summarized as follows:

- Graves’ disease, non-Graves hyperthyroidism, and thyroid storm
- Pre-resective embolization before thyroid surgery (goiter or thyroid cancer)
- Embolization for palliative purposes in goiter and thyroid cancers with an intrathoracic extension (cervicomediastinal)

In the treatment of thyroid nodules, TAE is mostly used for nodules that are large in size. Research suggests that ablation techniques are less effective or take a long procedural time in nodules with a volume above 20-30 ml (75). In addition, the presence of multiple nodules and the cervicomediastinal extension of nodules make ablation techniques difficult and reduce their success rates. These conditions also present challenges for surgery and increase the risk of complications (26). In a study conducted by Yılmaz et al., it was stated that TAE alone could be an alternative to surgery for retrosternal goiter, as it reduced intrathoracic extension by 50% and provided symptomatic improvement (26). Therefore, multiple and large thyroid nodules and those with a cervicomediastinal extension represent clinical cases in which TAE may be superior to TA and surgery. In such cases, RAI is a viable treatment method that can be applied. However, RAI treatment affects the entire thyroid gland, frequently resulting in the development of hypothyroidism. In contrast, the TAE treatment is patient-specific. Embolization is performed on a single artery in the treatment of solitary nodules, while embolization of two or three arteries is required for the treatment of Graves’s disease and MNG. Thus, the thyroid lobe, which is normal or less affected compared to the other lobe, remains partially or completely intact (26). Therefore, it is emphasized that the risk of hypothyroidism is reduced compared to RAI treatment. In a study conducted by Yılmaz et al., there was an approximately 56% reduction in thyroid gland volume with TAE, and the authors emphasized that this success rate was at least equivalent to that of RAI (26, 77). TAE is a good alternative in cases where standard treatments cannot be used in the treatment of thyroid nodules, such as a lack of response to RAI, intolerance to anti-thyroid drugs, and contraindications for surgery (78).

Various liquid embolizing agents, including ethanol, histoacryl + lipiodol^®^ combination, embosphere^®^, and PVA, have been used in different studies on TAE (78, 79). Brzozowski et al. reported that the use of histoacryl + lipiodol could result in the persistence or recurrence of hyperthyroidism due to the high iodine content in lipiodol (78). The most frequently used agent in the literature is standard PVA particles. While there is no agreement across studies concerning particle size, particles ranging from 150 to 700 µm have been employed. Although the use of small particles has the advantage of inducing more significant ischemia and necrosis, there is a risk of non-target embolization as they may separate from the target tissue through arterioles and veins. On the other hand, it is known that the efficacy of embolization may diminish when the particle size is increased (80). Similar to the case of RAI treatment, temporary hyperthyroidism may occur after TAE. This is considered to arise from the migration of thyroid hormones into the circulation from necrotic thyroid tissue. Although this is a potentially dangerous situation, especially in elderly patients with comorbidities, no major problem associated with post-TAE thyrotoxicosis has been described in the literature. A recent study reported that patients who developed thyrotoxicosis after TAE were asymptomatic or had mild symptoms; therefore, they mostly did not require treatment. In symptomatic patients, anti-thyroid drugs (methimazole and propylthiouracil) and cholestyramine, an agent that inhibits the intestinal reabsorption of thyroid hormones, have been used. Research has highlighted that cholestyramine may be more effective than anti-thyroid drugs due to the underlying cause being excessive hormone release into the bloodstream (26).

In the literature, TAE-related complications are reported separately in each study. Therefore, it would be appropriate to use the SIR guidelines as a basis to group these complications as minor or major. Life-threatening complications, those that cause a prolonged hospital stay, significant morbidity, or disability, and those resulting in permanent sequelae should be considered major (81). Among the documented complications are neck pain, groin hematoma, hyperthyroidism crisis, transient hypocalcemia, post-embolization syndrome, and radial artery spasm (for the transradial technique) (26, 75, 78, 82, 83). The major and minor complication categories of groin hematoma and hyperthyroidism crises vary across the existing studies. This appears to be due to the different classifications made depending on clinical severity and the need for intervention according to the SIR terminology. It is also important to address the risk of neurological and non-neurological complications due to non-target embolization, although, to the best of our knowledge, this has not yet been reported in the literature (84).

There are limited studies in the literature on the use of TAE in the treatment of solitary thyroid nodules and MNG. Yılmaz et al. achieved a 69% reduction in nodule volume in solitary or dominant nodules, a 56% reduction in gland volume in patients with MNG, and a 50% reduction in the presence of retrosternal goiter with an intrathoracic extension. The authors observed that all patients who were euthyroid before the procedure remained euthyroid after the procedure, while 86% of the patients with non-Graves hyperthyroidism became euthyroid in the sixth month after the procedure (26). In another study conducted on large-sized, benign solitary and symptomatic nodules, it was found that the nodule size decreased by approximately 55% within the first month and around 82% in the third month of follow-up (75).

TAE is also utilized in the treatment of malignant thyroid nodules. The two important uses of embolization in thyroid cancer, as shown in the literature, are to reduce pre-operative gland blood flow and palliation for symptoms related to thyroid cancer. In a study undertaken by Dedecjus et al., both superior thyroid arteries were embolized with PVA + coil, and one inferior thyroid artery was embolized with PVA alone. The authors determined that this approach shortened the operation time and reduced blood loss during the procedure. However, since there was a massive increase in the thyroglobulin level after pre-resective embolization, it was recommended that the operation be performed within 36 hours (83). Ramos et al. performed pre-resective TAE with histoacryl + PVA on three arteries (two superior and one inferior thyroid artery) in a patient with symptomatic cervicomediastinal goiter and PTC metastasis in the spine. They emphasized that in the operation performed seven days after the procedure, the blood supply and size of the gland decreased, which facilitated the surgery (85). For a similar purpose, Rulli et al. applied TAE with PVA to bilateral superior thyroid arteries in a patient diagnosed with thyroid lymphoma and stated that this procedure, performed 48 hours before the operation, minimized intraoperative bleeding (86). Tracheal invasion of anaplastic and medullary thyroid cancer causes problems such as pain and hemorrhage that significantly reduce patients’ quality of life. Previous literature has documented successful embolization procedures performed for palliative purposes in this group of patients (87, 88).

Although chemoembolization and radioembolization do not yet have established clinical use for thyroid cancer, they are also promising. Chemoembolization with drug-eluting beads loaded with doxorubicin (DEB-DOX) and radioembolization with the locoregional administration of antibody-bound radio-immunotherapeutic agents (e.g., Yttrium-90-anti-CEA) are considered to be potentially beneficial in the treatment of medullary thyroid cancer. Dabrafenib is a targeted drug that has been shown to be effective in advanced PTC when used systemically. Recently, the i-Dabrafenib form, which can bind to drug-loaded particles, has been developed. This has led to the idea that radiosensitization and chemosensitization could be achieved in advanced-stage PTC through embolization performed in the form of DEB-DAB (84).

## 4 Conclusion

In conclusion, interventional radiology has an important place in the diagnosis and treatment of thyroid nodules and will be able to offer unique insights to endocrinologists and surgeons in terms of various aspects of patient management in the future.
